# Supplementary material for: Potential Risk, Spatial Distribution, and Soil Identification of Potentially Toxic Elements in Lycium barbarum L. (Wolfberry) Fruits and Soil System in Ningxia, China
Source: Int J Environ Res Public Health. 2022 Dec 3;19(23):16186. doi: 10.3390/ijerph192316186 (PMC9739834; doi:10.3390/ijerph192316186)
Supplement: Supplementary file 1 [file ijerph-19-16186-s001.zip › ijerph-1990418-supplementary.pdf]

Supplementary Material

Potential Risk, Spatial Distribution, and Soil Identification of Potentially Toxic Elements in the *Lycium barbarum* L. (Wolfberry) Fruits and Soil System in Ningxia, China

Tongning Zhou [a], Yan wang[a], Jiaqi qin[a], Siyuan Zhao[a], Deyan Cao[a], Meilin Zhu [a, b*],

Yanxue Jiang [c*],

[a] College of Public Health and Management, Ningxia Medical University, Yinchuan 750004, China

[b] College of Basic Medical Sciences, Ningxia Medical University, Yinchuan 750004,

[c] College of Environment and Ecology, Chongqing University, Chongqing, 400045,

* Corresponding author：

E-mail: jay70281@163.com (Meilin Zhu);

jiangyanxue@cqu.edu.cn (Yanxue Jiang)

Table S1. Methods of PTEs and physicochemical properties determination

	Method	[a]Basis	Units	Detection limit
Cr	Soil and sediment—Determination of copper, zinc, lead, nickel and chromium—Flame atomic absorption spectrophotometry	HJ 491-2019	mg/kg	4
As	Soil quality—Analysis of total mercury,arsenic and lead contents—Atomic fluorescence spectrometry—Part 2:Analysis of total arsenic contents in soil	GB/T 22105.2-2008	mg/kg	0.01
Cd	Soil quality—Determination of lead,cadmium—Graphite furnace atomic absorption spectrophotometry	GB/T 17141-1997	mg/kg	0.01
Pb	Soil and sediment—Determination of copper, zinc, lead, nickel and chromium—Flame atomic absorption spectrophotometry	HJ 491-2019	mg/kg	10
Hg	Soil quality—Analysis of total mercury,arsenic and lead contents—Atomic fluorescence spectrometry—Part 1:Analysis of total mercury contents in soil	GB/T 22105.1-2008	mg/kg	0.002
Cu	Soil and sediment—Determination of copper, zinc, lead, nickel and chromium—Flame atomic absorption spectrophotometry	HJ 491-2019	mg/kg	1
Zn	Soil and sediment—Determination of copper, zinc, lead, nickel and chromium—Flame atomic absorption spectrophotometry	HJ 491-2019	mg/kg	1
Ni	Soil and sediment—Determination of copper, zinc, lead, nickel and chromium—Flame atomic absorption spectrophotometry	HJ 491-2019	mg/kg	3
pH	Soil-Determination of pH-Potentiometry	HJ 962-2018	-	-
SOM	Soil Testing—Part 6: Method for determination of soil organic matter	NY/T1121.6-2006	g/kg	-
CEC	Soil quality—Determination of cation exchange capacity（CEC）—Hexamminecobalt trichloride solution-Spectrophotometric method	HJ 889-2017	coml^{+}/kg	0.8

[a] From *the agricultural industry standards of the people's Republic of China*, *the national standards of the people's Republic of China* and *the national environmental protection standards of the people's Republic of China*.

Table S2. Contamination degree of potential ecological risk index

RI	E_r	Risk level
RI <150	E_r<40	Low
150≤ RI <300	40≤E_r<80	Medium
300≤ RI <600	80≤E_r<160	Heavy
RI ≥600	160≤E_r<320	High
	E_r≥320	Serious

Table S3. Contamination degree of enrichment factor

EF	Contamination Degree
<2	No or minimal pollution
2–5	Moderate pollution
5–20	Significant pollution
20–40	Strong pollution
>40	Extremely strong pollution

Table S4. Pearson correlations between PTEs content in wolfberry and soil indicators

In wolfberry \ In soil		Ni	Cu	Zn	As	Pb	Cr	Hg	pH	SOM	CEC
Ni	Pearson Correlation	-0.246	-0.026	-0.221	0.249	0.06	0.199	-0.069	-0.071	0.308	0.089
	[a]Sig.	0.143	0.881	0.189	0.137	0.723	0.237	0.686	0.677	0.063	0.599
Cu	Pearson Correlation	-0.065	0.225	-0.099	[b]**0.377**	0.212	-0.163	0.268	-0.029	0.129	[c] **0.441**
	[a]Sig.	0.704	0.181	0.56	0.022	0.208	0.334	0.108	0.864	0.447	0.006
Zn	Pearson Correlation	0.131	0.073	-0.137	0.122	0.122	-0.161	0.07	0	0.189	[b] **0.346**
	[a]Sig.	0.439	0.669	0.419	0.473	0.471	0.341	0.679	0.999	0.263	0.036
As	Pearson Correlation	0.247	-0.136	-0.241	-0.154	-0.046	0.183	-0.022	0.185	0.275	0.128
	[a]Sig.	0.14	0.421	0.151	0.362	0.786	0.277	0.897	0.272	0.1	0.452
Cd	Pearson Correlation	-0.019	-0.172	-0.133	-0.094	-0.055	-0.058	0.092	0.222	0.179	0.018
	[a]Sig.	0.911	0.309	0.431	0.58	0.747	0.735	0.589	0.186	0.289	0.916
Pb	Pearson Correlation	-0.312	0.092	-0.28	-0.162	0.099	-0.092	-0.204	[b] **0.358**	0.198	-0.042
	[a]Sig.	0.06	0.588	0.093	0.339	0.559	0.589	0.225	0.029	0.24	0.807
Cr	Pearson Correlation	0.078	0.096	0.015	0.043	0.276	-0.073	0.32	-0.018	0.075	0.275
	[a]Sig.	0.645	0.573	0.929	0.802	0.098	0.668	0.053	0.916	0.659	0.099

[a] Sig. means significance.

[b] Correlation is significant at the 0.05 level (2-tailed).

[c] Correlation is significant at the 0.01 level (2-tailed).

Table S5. Fitting of semivariogram parameters for Kriging interpolation of soil PTEs content

	Model Type	Nugget C_0	Sill $C+C_0$	[a]Proportion $C_0/(C+C_0)$	R^2	range major	range minor	range
Ni anisotropic variograms	Gaussian	71.4	336	0.213	0.442	99731	99731	
Cu anisotropic variograms	Gaussian	0.111	0.837	0.133	0.492	791547	791547	
Zn isotropic variograms	Spherical	1.50	49.4	0.0300	0.676			14690
As anisotropic variograms	Gaussian	1.95	7.09	0.276	0.398	810946	99420	
Pb isotropic variograms	Spherical	0.0800	6.85	0.0120	0.433			6870
Cr isotropic variograms	Spherical	0.00630	0.243	0.0260	0.879			31790
Hg isotropic variograms	Gaussian	24.3	120	0.203	0.381			94068

[a] $C_0/(C+C_0)$ is the ratio of the spatial variation caused by the random part to the total spatial variability. This is an important parameter for expressing the spatial autocorrelation of regionalized variables. When $C_0/(C+C_0) > 0.75$, the PTEs contents have a weak spatial correlation. When $C_0/(C+C_0)$ is between 0.25 and 0.75, the PTEs contents have a medium spatial correlation. When $C_0/(C+C_0) < 0.25$, the PTEs contents have a strong spatial correlation.

Table S6. Fitting of semivariogram parameters of PTEs in wolfberries

	Model Type	Nugget (C_0)	Sill ($C+C_0$)	Proportion ($C_0/(C+C_0)$)	R^2	Range major	Range minor	Range
Ni (isotropic variograms)	Spherical	0.0134	0.0646	0.207	0.63	-	-	81100
Cu (isotropic variograms)	Exponential	2.08	7.493	0.278	0.298	-	-	243300
Zn (isotropic variograms)	Spherical	1.5	49.39	0.030	0.676	-	-	14690
As (isotropic variograms)	Spherical	0.1502	0.4164	0.361	0.712	-	-	35560
Pb (anisotropic variograms)	Gaussian	0.0274	0.1031	0.266	0.224	9086338.5	9086338.5	-
Cd (anisotropic variograms)	Gaussian	0.000287	0.000813	0.353	0.173	9088070.6	9088070.6	-
Cr (anisotropic variograms)	Gaussian	0.015	0.059692	0.251	0.305	763314.8	224473.8	-
